# A Case of Monstrous Birth

**Published:** 1790

**Authors:** Richard Dinmore

**Affiliations:** Surgeon at Watton in Norfolk


III. *A Cafe of monftrous Birth. By Mr.* Richard Dinmore, *Surgeon at Watton in Norfolk. Communicated to Dr.* Simmons *by* Andrew Marfhal, *M. D. Member of the College of Phyficians, and Reader of Anatomy in London.*

ON the 20th of June, 1789, I was fent for by the officers of a neighbouring parifh to a poor woman who had been feveral hours in labour. A midwife was attending; but the prefentation being uncommon, fhe had defired a furgeon might be called in to affift her. Upon examination I felt, without the os externum, a firm fubftance, which I afterwards found to be liver; the next pain forced out in-

 teftines; teſtines; and immediately after the body of a child, of an unuſual appearance, followed.

The membranes (which, in order to convey a diſtinct idea of the whole birth, are ſuppoſed not to be ruptured) contained the liquor amnii, with a monſtrous child all but the abdomen; the placenta, as uſual, exterior. The child was truncated at the lower end of the dorſal vertebræ, the lumbar vertebræ and baſis of the trunk being wanting. The extremities were folded up, applied cloſe to the monſtrous trunk; the upper extremities, which were connected (in the uſual way) to the upper lateral parts of the thorax, were turned up to the ears; the right lower extremity, growing from the lower end of the back bone before, was applied cloſe up to the breaſt; the left lower extremity, or what was inſtead of it, grew from about the laſt dorſal vertebra behind, and was applied upwards along the back.

The funis, ariſing from the placenta, was unuſually ſhort: it could not be diſtinguiſhed by the epithet *umbilicalis*, for it paſſed into the right hypochondrium, to the cava inferior, at the diaphragm, being exterior to, rather than inveſted by, the membranes. The membranes themſelves terminated in the edge of the cuticle of of the monſtrous child all round the lower end of the deficient trunk.

As to the abdomen, it hung as a large ſtrong bag, from the abrupt edge of the *cutis vera* of the child, around the lower end of its imperfect trunk, adhering exteriorly to the chorion by its mouth or upper part, ſo that it appeared to depend alſo from the membranes: it was the naked peritonæum, or a ſac ſimilar to the peritonæum, and contained abdominal viſcera in the ſame way the peritonæum does. The contents were a ſtomach, with ſmall inteſtines; and there being no great inteſtines, the inteſtinum ilium turned up where the right flank might have been, and becoming altogether exterior to the ſac, paſſed into the right axilla of the child within the cavity of the membranes, terminating by a ſmall ſkin hole, which ſeemed to be the anus.

The liver, which was large, and ſomewhat misſhapen; a ſpleen, with unuſual appendages; one ſingle kidney, misſhapen, and placed central among the convolutions of the inteſtines; capſulæ renales, ſingle, and ſeparated from the kidnies, and ſituated in the uſual ſeats of the kidnies; a bladder of urine, ſituated below the ſingle kidney, and without ureters; a very misſhapen misſhapen uterus placed below the bladder, and having a long vagina continued from it, which was turned up along with the inteſtinum ilium till it alſo deſerted the peritonæum, and paſſing to within the membranes, terminated alſo in the right axilla, by a ſmall hole, contiguous nearly to the anus. This other ſmall hole was perhaps a miſplaced and misſhapen vulva. The child, thus monſtrous, ſeemed to be a female.

Such was the monſtrous birth, conſidered as an entire whole.

I would not pretend to fix what phyſiological concluſions might be deduced from the above account. One concluſion, a practical one, is obvious; that as monſtrous births of this ſort do actually occur, it ſhould ſuſpend alarm of danger when, in delivery, inteſtines preſent themſelves, *propriâ formâ*, to the fingers or view of the midwife.

*Watton, Norfolk,*

Auguſt 12, 1790.

IV. *Caſe*

